# Homer-3 antibody in autoimmune cerebellar syndromes: a potentially treatable mimic of MSA-C – A review

**DOI:** 10.3389/fimmu.2025.1660587

**Published:** 2025-08-13

**Authors:** Patryk Chunowski, Natalia Madetko-Alster, Piotr Alster

**Affiliations:** Department of Neurology, Medical University of Warsaw, Warsaw, Poland

**Keywords:** Homer-3 antibodies, autoimmune cerebellar ataxia, MSA-C, APS, neurodegeneration

## Abstract

Autoimmune cerebellar ataxia (ACA) associated with anti-Homer-3 antibodies is a rare but increasingly recognized immune-mediated neurological condition. It represents a potentially treatable cause of sporadic cerebellar syndrome and may clinically mimic primarily multiple system atrophy of the cerebellar type (MSA-C), and less frequently, other atypical parkinsonian disorders. Because of the significant clinical overlap with neurodegenerative diseases, particularly MSA-C, Homer-3–associated ACA may be underdiagnosed or misdiagnosed, delaying effective treatment. This narrative review synthesizes the currently available literature on anti-Homer-3 immunoglobulins, with a focus on their pathophysiological role, diagnostic utility, therapeutic response, and clinical differentiation from neurodegenerative conditions. Homer-3 is a postsynaptic scaffold protein expressed in Purkinje cells, where it plays a key role in calcium signaling through metabotropic glutamate receptor pathways. Antibodies against Homer-3 have been identified in patients with a wide range of neurological and neuropsychiatric symptoms, most commonly with subacute cerebellar ataxia. Neuroimaging in such cases frequently shows cerebellar atrophy or inflammation, and cerebrospinal fluid analysis often reveals inflammatory markers. The treatment with immunotherapy, particularly corticosteroids and intravenous immunoglobulins, which has showed encouraging results, however therapeutic outcomes can vary. The aim of this review is to collect and analyze all currently available data on anti-Homer-3–associated ACA, with the goal of raising clinical awareness and emphasizing the importance of early recognition and aggressive intervention.

## Introduction

Ataxia is a neurological syndrome marked by impaired coordination, which in the majority of cases results from cerebellar dysfunction. It typically presents with unsteady gait, poor limb coordination, slurred speech, and involuntary eye movements (nystagmus) (1). Importantly, ataxia is not a diagnosis in itself but rather a clinical sign that warrants further investigation to determine the underlying cause. Although rare, ataxias can stem from a range of conditions, broadly categorized as either genetic or acquired (2, 3). The genetic basis of ataxia is well established, a positive family history directs the diagnostic approach toward genetic forms of ataxia, including autosomal dominant spinocerebellar ataxias (SCAs). [Chien] Inherited cerebellar ataxias affecting approximately 1 in 10,000 individuals (4). The causes of acquired ataxia include toxins, infections, vitamin deficiency, vascular disturbances, tumors alongside with paraneoplastic syndromes and immune mediated disorders (5). A representative example of acquired ataxia is autoimmune cerebellar ataxia (ACA), a significant and potentially treatable cause of sporadic cerebellar syndromes (6). ACA is a notable subtype of cerebellar ataxia driven by immune mediated mechanisms. As research in this area grows, a more detailed understanding of the pathophysiology and clinical features of ACA is emerging. Over time, the clinical understanding of primary autoimmune cerebellar ataxia (PACA) has significantly improved and it has been classified as a distinct clinical entity. PACA is characterized by an autoimmune response directed against cerebellar neurons, occurring without a known external trigger. Studies have revealed a high prevalence of autoimmune conditions among patients with idiopathic sporadic ataxias. For instance, approximately 60% of these patients tested positive for anti-cerebellar antibodies (2) The multiple system atrophy (MSA) emerged as the most prevalent diagnosis among individuals with cerebellar ataxia. In the study conducted by Kudo et al. 93 of the 243 patients has been diagnosed as a MSA (7). MSA belongs to the group of synucleinopathies. It is a rapidly progressing disorder, classified into two primary forms: the parkinsonian type (MSA-P) and the cerebellar type (MSA-C) (8). Cerebellar symptoms such as gait ataxia, limb ataxia, dysarthria, and nystagmus are key features of MSA-C. Another disease that can mimic cerebellar symptoms and belongs to the group of atypical parkinsonian syndromes is progressive supranuclear palsy with predominant cerebellar ataxia (PSP-C) (9). Differentiating these symptoms from those of other conditions can be difficult and it is often misdiagnosed (8). Among the autoimmune cerebellar disorders, those associated with specific antibodies have drawn particular interest. In recent years, antibodies such as anti-Homer-3 have been frequently linked to ACA, highlighting a growing focus on antibody-mediated cerebellar syndromes (10–12).

## Aim of the review

Aim of the review is to collect all available data about anti-Homer 3 immunoglobulins in identifying immune mediated causes of cerebellar ataxia, which appears to be potentially treatable. This underscores the importance of careful differential diagnosis, particularly in the context of overlapping clinical features with atypical parkinsonian syndromes, and in particular with MSA-C.

## Homer-3 patophysiology

Encoded by *Homer1, Homer2* and *Homer3* genes (13) Homer proteins were mainly localized in the dendritic spines of Purkinje cells (14). Homer-3, a scaffold protein abundantly expressed in Purkinje cells and it is involved in regulating neuronal activity. The expression of Homer-3 is dependent on mTORC1 signaling (15). Excessive activation of mTORC1 triggered significant apoptosis in Purkinje cells and was associated with disrupted cellular homeostasis, including activation of a pseudohypoxic response, increased mitochondrial respiratory activity, and cell swelling (16). These proteins constitute a family of small adaptor molecules localized at postsynaptic densities (PSDs), where they facilitate the interaction between cytoplasmic domains of group I metabotropic glutamate receptors (mGluRIs) and other PSD-associated proteins, such as Shank, or membrane proteins like Ins(1,4,5)P3 receptors. Homer proteins regulate the membrane localization and postsynaptic organization of group I mGluRIs and modulate their coupling to G proteins as well as to intracellular Ca²^+^ stores mediated by Ins(1,4,5)P_3_ and ryanodine receptors (17).

## Diagnostics

Many antibodies are associated with ACA. The anti-Homer-3 antibody is one of them (18) and is increasingly reported finding in the literature. To best of our knowledge, 15 cases of Homer-3 antibody–associated autoimmune cerebellar ataxia have been reported. The average patient age was 44.5 years, with a female predominance (10 females and 5 males). Majority of the patients described below presented with prodromal symptoms upon admission, primarily in the form of vertigo-type dizziness or general dizziness accompanied by nausea. Less frequently observed prodromal symptoms in cases of ACA with positive Homer-3 antibodies included drowsiness (19), fever (12) sleep disturbances, concentration complaints or affect changes (20) In Liu et al. study Homer-3 antibodies were found in six patients (four women and two men). The median onset age was 54.5 years, however one adolescent was also affected. Most had subacute onset, and symptoms included cerebellar ataxia, encephalopathy with myeloradiculopathy, and rapid eye movement (REM) sleep behavior disorder (RBD) with autonomic dysfunction. All patients showed cerebellar syndrome with symptoms like dizziness, unsteady gait, limb ataxia, dysarthria, and nystagmus. Two patients exhibited encephalopathy with cognitive and motor impairments, with electromyography (EMG) findings suggesting denervation. Two others showed MSA-C-like features, including addtionally dysautonomia and RBD, with cerebellar syndrome reflecting cerebellar atrophy in three cases. Brain magnetic resonance imaging (MRI) revealed also other symmetrical cerebellar abnormalities hot cross bun sign and pontine atrophy. Lumbar puncture revealed leukocytosis, elevated protein, or oligoclonal bands in some cases (12). In the case of A 38 year old male, who developed symptoms similar to the previously described case, additionally exhibited a partial seizure with secondary generalization and a papilloedema. Due to the suspicion of a neuroinfection, a lumbar puncture was performed, which revealed a high opening pressure, elevated protein concentration with mild lymphocytic pleocytosis (60/µL, 78% lymphocytes) The brain MRI was initially normal, however revealed a mild cerebellar atrophy in follow-up (19). In a different report of a 65-year-old woman with a typical prodromal symptoms and typical disturbances in neurological examination for Homer-3 positive ACA. In a CSF detected in addition to leukocytosis, an increased level, approximately double the norm of IgG index. The patient did not show improvement following steroid therapy. Homer 3 was identified as an autoantigen associated with the immune response in idiopathic subacute cerebellar ataxia (21). A 56 years old woman was admitted to the hospital due to severe mental disturbances comprising sleep, memory, concentration and mood complaints. Notably, this patient did not present with either typical prodromal symptoms or cerebellar syndrome characteristic of individuals with positive anti-Homer-3 antibodies. Phosphorylated tau protein 181 was found to be elevated in the lumbar puncture. Due to the presence of phosphorylated tau protein 181 in the CSF, along with rapidly progressing cognitive and affective symptoms, a suspicion of Alzheimer’s disease (AD) was raised. However, in the absence of laboratory findings (including no reduction in the Aß42/40 ratio or decreased Aß42 level in the cerebrospinal fluid) and given the symptoms suggestive of a cerebellar origin (due to cerebellar atrophy in MRI), AD was not diagnosed (20). A 25-year-old man with a typical clinical presentation. Neurological examination revealed dysmetria and nystagmus. MRI showed cerebellar hyperintensities, and CSF analysis indicated inflammation. Serum tested positive for Homer-3 antibodies. Despite steroid treatment, his symptoms worsened, including severe speech impairment and psychiatric symptom such as difficulties with emotional control. Months later, he relapsed with intention tremor, lower limb weakness, and worsening cerebellar lesions on MRI. High-dose steroids and further plasma exchanges were ineffective, prompting rituximab administration. Gradual clinical improvement followed, with notable reduction in tremor and limb weakness, although gait remained unstable (22). A 58 year old female with distinctive clinical picture for Homer-3 positive ACA, except for marked vision disturbances likely resulting from nystagmus. Of note cranial MRI did not show cerebellar atrophy, a typical finding in cerebellar degeneration. Initial autoimmune antibody screening returned negative, leaving the underlying cause unclear. However, anti-Homer-3 antibodies were later detected in both serum and cerebrospinal fluid using indirect immunofluorescence (11). A 10-year-old male was evaluated for a two-week history of cognitive decline, irritability, slurred speech, and cautious gait, indicating possible neurological dysfunction and coordination problems. Neurological examination revealed features of pan-cerebellar syndrome, including bradypsychia, dysarthria, and ataxia. Brain MRI showed hyperintensities in both cerebellar hemispheres and the vermis, suggesting cerebellar inflammation, atrophy has been intensifying as the disease advances. Anti-Homer-3 antibodies were detected in CSF but not in serum (23). The head tremor can be a Homer-3 positive ACA prodromal symptom (10, 24). This manifestation was observed in a prodromal disease course in a 20 year old female was uncontrollable head tremors accompanied by a bilateral horizontal nystagmus and a gait ataxia two days after a common cold. Brain MRI and CSF presentation with the positive oligoclonal banding pattern was indicative for Anti-Homer-3 ACA, which was confirmed in both CSF and serum (10). In another research, a tremor of the head was evident as well. Evaluation of the lower extremities revealed reduced distal muscle strength with no deep tendon reflexes. Serum testing revealed the presence of anti-Homer-3 antibodies (24). The next analysis of a 26 year old female showed marked cerebellar oedema with contrast enhancement, indicating cerebellar inflammation connected with Homer-3 antibodies presence. The patient was treated with intensive immunosuppressive therapy; however, her clinical response was limited. A follow-up MRI two months later revealed cerebellar atrophy, reflecting likely irreversible cerebellar damage and suggesting a poor prognosis with potential long-term impacts on recovery and quality of life (25). It should be noted that the cited articles describe individual case reports, with the largest group consisting of six individuals with positive Homer-3 antibodies. However, the described cases are largely consistent. In another study, 59 patients of European descent from two German biobank cohorts, diagnosed with possible or probable MSA according to the Gilman criteria. Given that the reported cases of ACA with positive Homer-3 antibodies mimicking MSA-C are potentially treatable, individuals were screened for Homer-3 antibodies using a research-grade assay. Of these, 33 presented with the MSA-C phenotype and 26 with the MSA-P phenotype. Homer-3 antibodies were not detected in the serum of any patient (26). Considering that the previously cited case reports mainly involved the Asian population, there is a possibility of geographic variation in the prevalence of Homer-3 antibodies. Given the low prevalence of positive Homer-3 antibodies, a statistically significant impact of false-positive results cannot be excluded. This effect may arise from secondary immunoglobulin production or from differences in antibody detection methodologies or variability in the sensitivity and specificity of reagents used across laboratories (26).

## Treatment

Initial immunotherapy, typically involving high-dose intravenous corticosteroids, was the most commonly employed first-line intervention across nearly all cases. For example, in the case series by Liu et al. the majority experienced clinical improvement following immunotherapy, implying corticosteroids and/or intravenous immunoglobulins (IVIG). 4 patients exhibited partial clinical improvement, specifically in ataxia, motor weakness, and encephalopathy, 1 patient achieved stabilization of their symptoms. The last patient continued to worsen despite treatment, suggesting drug resistant disease. 2 patients who initially improved experienced clinical relapse during corticosteroid tapering or following the discontinuation of IVIg indicating that the disease tends to relapse when immunosuppressive treatment is reduced or stopped. Radiologically, patients 3 and 6 showed shrinkage of cerebral lesions on MRI, suggesting at least partial responsiveness of the underlying inflammatory process to treatment. However, serum Homer-3 antibodies remained detectable in patients 1, 2, and 3 at their final follow-up, indicating persistent immunological activity despite clinical management (12). A 38-year-old man described by Höftberger was treated with intravenous steroids and immunoglobulins, followed by long-term low-dose oral prednisone (7.5 mg daily for two years), which led to partial recovery. The individual remained independent in daily activities with only mild residual symptoms. Post-treatment brain MRI to assess central nervous system (CNS) morphological changes was not performed in this case (19) Other cases reported varied responsiveness to corticosteroids. A 65-year-old woman in the Zuliani study failed to respond to steroid therapy, despite laboratory confirmation of anti-Homer-3 autoimmunity and clear cerebellar involvement, highlighting a subset of patients who may be steroid-resistant. In contrast, a 10-year-old male described by Kuang et al. initially showed no improvement with corticosteroids but experienced gradual recovery after a course of intravenous immunoglobulin (IVIG), achieving almost complete resolution of symptoms over a year (21). Combination immunotherapy proved especially effective in more severe or relapsing conditions. A 25 years old man exhibited worsening symptoms despite steroid treatment. He improved the treatment by following plasma exchange, but later the symptoms recurrent. High-dose steroids and repeated plasmapheresis failed to control the relapse. It was decided to the escalate the therapy to rituximab which is referred as a B-cell depleting therapy, which led to gradual symptom resolution (22). Similarly, in one reported case, a 58 years old woman underwent a multi-modal approach: high-dose methylprednisolone, 5 plasmapheresis sessions, and additional chemotherapy (epirubicin and cyclophosphamide) along with IVIG, resulting in significant clinical improvement (11). In contrast, some cases demonstrated more rapid improvement. A 20 years old woman developed an acute cerebellitis following a viral infection, responded well to a combination of high-dose methylprednisolone and IVIG, followed by tapering steroids. She regained most coordination within weeks, although mild residual symptoms persisted (10). Not all the patients responded favorably. In a particularly severe case, a 26-year-old woman with cerebellar inflammation confirmed by MRI received intensive immunosuppression. Despite therapy, the disease progressed to cerebellar atrophy within two months (25). A rare clinical manifestation is primarily cognitive and affective disturbances and due to the individual’s denial for further diagnostics and treatment, there is no data regarding this particular manifestation of Homer-3 positive ACA. Impaired control of emotions is another psychiatric symptom which developed despite of steroid administration with temporary improvement following plasma exchange, later requiring administration of rituximab (22). The most common treatment outcome was partial improvement, typically following therapy with corticosteroids, IVIG, plasmapheresis, mycophenolate mofetil, or rituximab. While several patients showed significant improvement or clinical stabilization, others experienced limited benefit or deterioration, indicating considerable variability in treatment response in Homer-3 autoimmunity. The summary of the reported case studies has been presented in **Table 1**.

**Table 1:** Summary of magnetic resonance imaging (MRI) findings, cerebrospinal fluid (CSF) results, biological specimens, and antibody detection methods in patients with anti-Homer-3 positive autoimmune cerebellar ataxia (ACA).

Age	Sex (F/M)	Homer-3 antibody detection method	Biological material in which Homer-3 antibodies were investigated (+ or -)	MRI finding	CSF evaluation	Anti-Homer-3 positive ACA clinical symptoms	Symptoms overlapping with possible MSA-C	Implemented treatment	Treatment outcome
20	F	IIFT	Serum + CSF +	An increased signal in the right cerebellar hemisphere without enhancement	intracranial pressure elevation, pleocytosis, increased protein levels, and a positive oligoclonal band	CA, prodromal head tremor (10)	CA	High-dose methylprednisolone with gradual dose tapering. Prednisone. IVIG. Ganciclovir and MMF	Improvement with persisting mild residual symptoms (10)
58	F	IIFT	Serum + CSF +	No abnormalities	Lymphocytic pleocytosis of 11 cells/µl with an intrathecal IgG synthesis	CA, dysarthria (11)	CA, dysarthria	High-dose methylprednisolone. 5 plasmapheresis sessions. IVIG chemotherapy (epirubicin and cyclophosphamide) due to co-existing breast adenocarcinoma	Significant clinical improvement (11)
65	M	IIFT	Serum + CSF -	No abnormalities	The results are unreliable due to blood contamination in the CSF	CA, RBD, dysuria, postural hypotension (12).	CA, RBD, dysuria, postural hypotension	CS, IVIg and PLEX	Deterioration (12)
14	M	IIFT	Serum + CSF -	No abnormalities	Leukocytosis, elevated protein and oligoclonal bands were present	CA, prodromal fever, limb weakness and hyporeflexia, seizure psychosis (12)	CA	CS, IVIg	Partially improved with IVIg and CS with 2 relapses during CS tapering (12)
84	F	IIFT	Serum + CSF -	No abnormalities	Mild leukocytosis	CA (12)	CA	CS	Stabilization (12)
59	F	IIFT	Serum + CSF N/A	Hyperintensity in bilateral frontal and parietal cortex	No abnormalities	CA, limb weakness and hyporeflexia, cognitive impairment (12)	CA	IVIg, CS	IVIg (improvement followed by relapse) and CS (improvement) (12)
46	F	IIFT	Serum + CSF +	Cerebellum atrophy	Present oligoclonal bands	CA (12)	CA	CS, MMF	Partial improvement (12)
50	F	IIFT	Serum + CSF -	cerebellum and pons atrophy accompanied by hot cross bun sign	No abnormalities	CA, RBD, dysuria, postural hypotension. (12)	CA, RBD, dysuria, postural hypotension	CS, MMF	Partial improvement (12)
38	M	Western blot	Serum + CSF N/A	Initially without pathology after 10 months disease duration cerebellar atrophy was observed	Pleocytosis with elevated protein level	CA, dysarthria prodromal headache, nausea, vomiting, confusion with encephalopathy and myeloradiculopathy (19)	CA, dysarthria	Prednisone, IVIg followed by oral CS therapy	Partial improvement (19)
56	F	IFA	Serum + CSF +	Cerebellar atrophy.	elevated phosphorylated tau protein 181 (ptau 181)	Cognitive and affective disturbances (20)	–	Repetitive transcranial magnetic-stimulation treatment, with no further therapy	No improvement (20)
65	F	IIFT	Serum + CSF N/A	No abnormalities	Lymphocytosis with elevated IgG index	CA (21)	CA	Prednisone	Stabilization followed by cerebellar ataxia which developed years later (21)
25	M	IIFT	Serum + CSF -	Progressive cerebellar atrophy with T1 mild enhancement	Lymphocytosis with elevated IgG index. The protein concentration was elevated from the outset, with subsequent worsening alongside with clinical deterioration	CA, limb weakness, psychosis (22)	CA	Methyloprednisolone pulses followed by oral prednisolone, PEX, Rituximab	CS (dysarthria and psychosis deterioration), PEX (partial improvement), relapse treated with rituximab, with partial improvement (22)
10	M	IIFT	Serum – CSF +	Progressive cerebellar atrophy with multiple lesions	Granular leukocytosis	CA, dysarthria, cognitive impairment (23)	CA, dysarthria	CS, IVIg	CS (no improvement) IVIg (significant improvement) (23)
51	F	IFA	Serum + CSF +	Cerebellar atrophy. Hot cross Bun sign	Lymphocytic pleocytosis with intrathecal IgG synthesis	CA, head tremor, limb weakness with absence of deep tendon reflexes (24)	CA	CS, MMF	Partial improvement (24)
26	F	IHC	Serum + CSF +	Cerebellar oedema and contrast enhancement with subsequent cerebellar atrophy	N/A	CA (25)	CA (29)	Aggressive immunosuppression (undefined)	Limited improvement (25)

IIFT, indirect immunofluorescence technique; IFA, indirect immunofluorescence assay; IHC, immunohistochemistry; N/A, not available; CA, cerebellar ataxia; RBD, rapid eye movement sleep behavior disorder; CS, corticosteroid; IVIg, intavenous immunoglobulin; PLEX, plasma exchange; MMF, Mycophenolate Mofetil.

## Discussion

The pathogenic potential of anti-Homer-3 antibodies remains uncertain. As described, the intracellular localization of Homer 3 makes antibody dependent cell mediated or complement mediated cytotoxicity less likely. Due to the wide range of interactions and functions of Homer-3 within Purkinje cells, a functional impact of antibody binding remains conceivable. In particular, interference with the Homer-3 interaction with mGluR1a could increase constitutive mGluR1 activity, as indicated by spontaneous inositol phosphate formation and calcium-dependent channel activity observed in cellular models following Homer-3 knockdown (1). In the context of the unclear mechanism underlying Homer-3 antibody production, secondary generation of these antibodies as a consequence of neurodegeneration cannot be excluded. This possible functional role of the antibodies could account for the clinical heterogeneity observed in ACA. Individuals with ACA may present with a diverse range of neurological symptoms. ACA may initially follow a relapsing pattern of symptoms (6). Common clinical features include dizziness, nystagmus (horizontal, vertical, or both), limb ataxia, trunk ataxia, and occasionally dysarthria. Other potential symptoms of Homer-3 positive ACA include gait ataxia, dysmetria, vertigo (27) limb and head tremor (22) Moreover, the Homer-3 antibody may be associated with affective disorders, RBD, encephalopathy, cognitive decline, concentration and orientation deficits causing an autoimmune-based MCI accompanied by depressive symptoms. A common symptom is also headache accompanied by vomiting and nausea, along with progressive confusion. which may resemble the clinical picture of a neuroinfection. To perform differential diagnosis, a lumbar puncture is often conducted (12). This particular type of ACA can be diagnosed when IgG Homer-3 autoantibodies are detected in the CSF (19). In CSF an inflammatory reaction and in some cases also oligoclonal bands are present (12). The pathological process can impact both the cerebellum and the hippocampus in the context of memory and cognitive dysfunction. Homer-3 is a protein that regulates AMPAR signaling in the hippocampus (28). Autoantibodies targeting Homer-3 have been linked to neurological conditions like cerebellar ataxia, which may mimic the clinical presentation of other disorders such as MSA-C (27). Homer-3 antibody associated ACA, MSA-C and PSP-C share overlapping clinical features, however they show several key differences. According to MSA guidelines to diagnose clinically probable MSA one characteristic autonomic dysfunction in combination with features of parkinsonism or cerebellar syndrome (29). Gait ataxia and limb dysmetria are prominent and early features in Homer-3 ACA (12) which are the core features of the cerebellar syndrome criteria, required for diagnosis of probable or possible MSA-C. In Homer-3 ACA a head tremor and limb tremor are frequently observed. head tremor is very rare and typically suggests essential tremor or other differential diagnoses (30–32). In Homer-3 ACA, encephalopathy, might occur due to the autoimmune etiology in the central nervous system resulting in among others severe cognitive dysfunction. Less pronounced cognitive impairment in form of MCI is frequently observed, particularly affecting memory, concentration, and orientation. Depressive symptoms are also commonly reported. These neuropsychiatric features are not included in the diagnostic criteria for MSA, Moreover, dementia is included in the diagnostic criteria for MSA-C as an exclusion. RBD may occur in both of the previously mentioned disease entities (20, 29). MRI often reveals cerebellar atrophy (6, 25), and in some cases, combined atrophy of the cerebellum and pons (12). Oligoclonal bands have been reported in the CSF (24), and denervation in electromyography (EMG) (12); however, none of these findings are specific. ACA with positive anti-Homer-3 antibodies can also mimic PSP-C, which is important considering that patients with PSP-C have predominantly been identified in Asian countries, while this condition has been noted to be very rare in Western populations (9). This is consistent with the cited literature review, in which most reported cases originate from Asia. Due to its rarity, specific diagnostic criteria for PSP-C have not been established (33). It is worth noting that in one case with positive Homer-3 antibody, ataxia or other typical, clinical symptoms for this entity were not detected, instead, the condition manifested primarily through psychiatric symptoms such as cognitive impairment, memory problems, and sleep disturbances resembling AD. However, in MRI of the brain characteristic cerebellar atrophy for MSA-C was observed (20). The above description confirms the variability of symptoms among patients with positive Homer-3 antibodies. These antibodies are increasingly recognized in the study of autoimmune neurological disorders. The Homer-3 protein, mainly found in the cerebellum’s Purkinje cells, plays a key role in calcium signaling by interacting with metabotropic glutamate receptors such as mGluR1 and IP3R (34). Homer-3 is an antibody directed against Purkinje cells (PCs) causing ACA that is not associated with cancer. There are speculations that the Homer-3 antibody may be associated with lung cancer (35) however, the evidence supporting this link remains unconvincing. Other antibodies targeting PCs have been identified, which are associated with paraneoplastic neurological syndromes. These antibodies often correlate with specific tumor types and present with characteristic neurological symptoms and subcellular localization within Purkinje cells (36). Anti-Sj/ITPR-1 antibodies have been linked to a variable disease course, often lacking a consistent clinical pattern. Likewise, anti-Ca/ARHGAP26 antibodies are associated with a range of neurological manifestations, and in some cases, have been connected to the presence of an underlying tumor. Anti-MAG antibodies are generally observed in patients with chronic gait disturbances and neuropathy, although the ataxia arises from central nervous system involvement. On the other hand, anti-Septin-5 antibodies have been identified in chronic cerebellar syndromes that appear resistant to immunotherapy. Anti-neurochondrin antibodies have been described in patients with long-standing cerebellar or brainstem symptoms (2), while Adapter-related protein complex 3 beta-2 subunit (AP3B2) antibodies are more frequently associated with a subacute form of cerebellar ataxia (37). Anti-Homer-3 antibodies, though rare, have been found in individuals with subacute cerebellar ataxia and are gaining recognition as potential indicators of autoimmune cerebellar syndromes (2). This distinction is clinically significant, as individuals with Homer-3 autoantibodies may respond to immunotherapy, unlike those with MSA-C, where treatment options are often limited (12). The physiological relevance of mGluR1 and its signaling interactions, including with IP3 and calcium signaling pathways, underscores a diverse role in sustaining neuronal health and function. With ongoing research into their pharmacological modulation, mGluRs are emerging as promising targets for innovative therapeutic strategies aimed at addressing MSA-C, providing hope for enhanced treatment modalities in clinical practice (38). Autoimmune causes, associated with a wide range of antibody mediated neurological disorders, can mimic MSA-C and should be considered in the differential diagnosis (39). However, it should be noted that other antibodies targeting Purkinje cells (PCs) have been described, which also primarily cause a cerebellar syndrome in autoimmune cerebellar syndromes (36, 40) All of them are linked with cerebellar syndrome to different extents and they are associated with different cancer types. Among the other, this group comprise: Contactin-associated protein-like 2 (Caspr2) correlated with memory and affective disturbances (41), Glial fibrillary acidic protein (GFAP) potentially connected with thymoma and other more rare neoplasms (40) and it is postulated that this antibody presented in plasma has a potential in differentating SCA with MSA-C. GFAP exhibit higher level in individuals with spinocerebellar ataxia type 7 (SCA7) (42, 43). In this ataxia type many symptoms are common with Homer-3 ACA such as incoordination, gait impairment, dysmetria, dysdiadochokinesia, nystagmus, ocular motor disturbances, dysarthria, postural tremor, hyperreflexia, and peripheral neuropathy (44). however vision loss is a differentiating feature of these disorders (45, 46). Similar ACA linked to ITPR1 presents with similar symptoms. This antibody is related to an increased likelihood of neoplastic processes (35). Alongside this, anti-KLHL11 antibodies have also been associated with paraneoplastic presentations. Notably, among patients with anti-KLHL11 antibody positivity, ataxia is frequently accompanied by encephalopathy, and in the majority of reported cases (11 out of 13), a seminoma was also diagnosed (47). Due to a similar mechanism of action referring the regulation of group I mGluR membrane localization, postsynaptic signaling organization, and their coupling to intracellular calcium stores via IP_3_ and ryanodine receptors the clinical presentation of ACA with anti-mGluR1 antibodies should be differentiated in comparison to that seen in ACA with anti-Homer-3 antibodies. However anti-mGluR1 ACA was more frequently associated with neoplasms, cognitive and behavioral disturbances, or dysautonomia. It is also worth noting that cerebellar atrophy is typically less pronounced compared to ACA associated with anti-Homer-3 antibodies. Clinical features of ACA associated with mentioned antibodies has been concluded in **Table 2**.

**Table 2:** Summary of key symptoms in autoimmune cerebellar ataxia (ACA) associated with selected antibodies.

Selected antibodies associated with ACA	Characteristic clinical features
Anti-Homer-3	Subacute cerebellar ataxia, potential response to immunotherapy, more pronounced cerebellar atrophy than in other ACA types
Inositol 1,4,5-trisphosphate receptor type 1 antibodies (Anti-Sj/ITPR-1)	Variable disease course, no consistent clinical pattern, similar symptoms to Homer-3 ACA, potentially associated with neoplasms
ARHGAP26 antibodies (Anti-Ca/ARHGAP26)	Broad neurological manifestations, sometimes associated with tumors
Myelin-associated glycoprotein antibodies (Anti-MAG)	Chronic gait disturbances, neuropathy, ataxia resulting from CNS involvement
Septin-5 antibodies (Anti-Septin-5)	Chronic cerebellar syndromes, most often resistant to immunotherapy
Neurochondrin antibodies (Anti-neurochondrin)	Long-standing cerebellar or brainstem symptoms
Adaptor protein complex 3 beta-2 subunit antibodies (Anti-AP3B2)	More commonly associated with subacute onset of cerebellar ataxia
Contactin-associated protein-like 2 antibodies (Anti-Caspr2)	Memory and affective disturbances
Glial fibrillary acidic protein antibodies (Anti-GFAP)	Possibly linked to thymoma and other rare neoplasms
Kelch-like protein 11 antibodies (Anti-KLHL11)	Ataxia accompanied by encephalopathy, particularly regarding the seminoma
Metabotropic glutamate receptor 1 antibodies (Anti-mGluR1)	Frequently associated with neoplasms, cognitive and behavioral disturbances, dysautonomia; less cerebellar atrophy compared to Homer-3 antibody positive ACA

Notably, a less frequent symptom is cerebellar ataxia, which represents the main clinical feature of neurodegeneration associated with Homer-3 antibodies (39). Other types of autoimmune cerebellar ataxia may be potentially treatable as well. These include ataxias associated with anti-glutamic acid decarboxylase (GAD) antibodies, gluten ataxia, and neurological manifestations linked to Hashimoto’s encephalopathy (48). These antibodies show a strong association with cerebellar ataxia facilitating distinguish it from other forms of ataxia and neurological diseases (12). Progressive supranuclear palsy, corticobasal degeneration, and multiple system atrophy make up roughly 10% of neurodegenerative parkinsonism. There is considerable clinical overlap among these disorders, including features commonly regarded as specific to each condition (49). The Homer-3 antibody related syndrome supports already existing hypothesis regarding the mutual relationship between neuroinflammation and neurodegeneration. Investigating the precise mechanisms underlying this condition may help determine whether neuroinflammation initiates neurodegeneration or whether immune system activation in the central nervous system occurs as a reaction to the pathological proteins aggregation. Autoimmune disorders may not only mimic MSA but also resemble other neurodegenerative diseases (50–52). The antibody of potential particular clinical relevance is anti-IgLON5 antibodies. Anti-IgLON5 antibodies contribute to tau accumulation by disrupting cytoskeletal integrity and inducing p-tau deposition in neurons, leading to synaptic dysfunction and features resembling neurodegenerative tauopathies (53–57). Neurodegenerative disorders associated with anti-IgLON5 antibodies are frequently characterized by sleep behavior disorder and bulbar symptoms (39, 58), but anti-igLON5 disease symptoms has a wider range expressing a clinical picture overlapping with disorders such as Alzheimer’s disease (AD) (59, 60), corticobasal degeneration (CBD) (61) progressive supranuclear palsy (PSP) (62, 63). The comparison of the previously mentioned neurodegenerative diseases has been included in **Table 3**.

**Table 3:** Comparison of selected symptoms of Homer-3 antibodies positive ACA, Anti-IgLON5 Disease, progressive supranuclear palsy (PSP), multiple system atrophy - cerebellar type (MSA-C), corticobasal degeneration (CBD) and Alzheimer’s disease (AD).

Symptom Disease	Homer-3 antibodies positive ACA	Anti-IgLON5 disease	Progressive supranuclear palsy (PSP) (PSP-C does not have exclusive diagnostic criteria)	Alzheimer’s disease (AD)	Crticobasal degeneration (CBD)	MSA-C
Cerebellar ataxia	Main symptom	Less frequent (59)	+ (in PSP-C)	–	–	+
Sleep disorder	RBD (12)	RBD and non-RBD and parasomnias (39, 58)	+ (63)	difficulties in falling asleep, sleep disruption and altered circadian sleep-wake cycle (59)	–	RBD
Cognitive impairment	+ (Rare)	+ (Often)	+	Main symptom	+	–
Oculomotor disturbance	Nystagmus (12)	Vertical	Vertical	–	Antisaccade performance abnormality	Nystagmus
Dysarthria	+ (19)	–	+ (Rare)	–	+ (Rare)	+
Dizziness	+ (39)	–	–	–	–	–
Dysautonomia	+ (39)	+	–	–	–	+
Encephalopathy	+ (19)	–	–	–	–	–
Myeloradiculopathy	+ (19)	–	- (33)	–	–	–
Imitation of	MSA-C	PSP (50)		- (60)	- (61)	- (29)

PSP-C, progressive supranuclear palsy – cerebellar type; RBD, rapid eye movement behavioral disorder.

Homer-3 antibody associated ACA shows a promising response to immunotherapy, with many cases demonstrating favorable clinical outcomes, including improvement or stabilization of symptoms. These results highlight the potential treatability of the condition, especially when compared to neurodegenerative ataxias. However, treatment response remains diverse, with some patients showing only partial benefit or experiencing relapses, indicating variability in disease course and therapeutic efficacy (12). First-line treatment typically involves high-dose intravenous corticosteroids, with or without IVIG. Many reported cases show clinical improvement (12, 22) Relapses have been observed, particularly during corticosteroid reduction or after discontinuation of IVIG, suggesting steroid dependent and resistant to treatment forms of the disease. Radiological improvements, including shrinkage of inflammatory lesions on MRI, support a partial responsiveness of the inflammatory process to treatment. Nevertheless, the continued presence of Homer-3 antibodies in the serum or cerebrospinal fluid, even after clinical improvement, indicates persistent immunological activity and underscores the need for long term monitoring in order to detect potential relapse in early stage and administer prompt therapy (22). However, not all individuals respond adequately, in several cases describe persistent or worsening symptoms despite standard immunosuppressive therapy. The aggressive and combined immunotherapy, even targeting B cell mediated autoimmunity, may be necessary in resistant or progressive forms of the disease. In severe cases, the risk of irreversible neurological damage further emphasizes the importance of early and intensive treatment (6, 11, 19, 21, 22, 25). Younger patients, particularly children and young adults, often show more rapid and complete recovery, especially when treatment is initiated early. In contrast, older adults or those with advanced cerebellar involvement are more likely to experience incomplete recovery or progression to cerebellar atrophy, despite aggressive treatment (23). Relapses have been observed, particularly during corticosteroid reduction or after discontinuation of IVIG, suggesting steroid dependent and treatment-resistant and forms of the disease. Radiological improvements, including shrinkage of inflammatory lesions on MRI, support a partial responsiveness of the inflammatory process to treatment. Nevertheless, the continued presence of Homer-3 antibodies in the serum or cerebrospinal fluid, even after clinical improvement, indicates persistent immunological activity and underscores the need for long-term monitoring in order to detect potential relapse in early stage and administer prompt therapy (22). It is worth emphasizing that in cases of ACA Homer-3 positive, the antibodies are not detected in every instance and may only become detectable at a later stage (11) or they might be identifiable only in CSF (23). This indicates that a negative initial screening for Homer-3 antibodies does not exclude the disease, and testing of both serum and CSF should be repeated. However, the exact timeframe after symptom onset at which these antibodies become detectable remains unclear. According to one study the cerebellar atrophy may be preceded by edema (25). Taking into the consideration, the fact that cerebellar atrophy progresses alongside with disease progression (23, 25), possibly Homer-3 seroconversion might progress also.

## Limitations

The main limitation of the discussed studies is the very small number of individuals with positive anti Homer-3 antibodies with no post-mortem neuropathological confirmation. Epidemiological data and past medical history were often insufficient. Not all patients underwent the same set of diagnostic tests which increases the risk of obtaining both false positive and false negative results. The implemented therapy was inconsistent and heterogeneous, and the time from symptom onset to the treatment initiation varied among patients Some individuals were not evaluated during follow-up after treatment. Notably, only articles written in English were included.

## Conclusion

This narrative review highlights the critical importance of recognizing immune mediated etiologies in cerebellitis, particularly in the presence of specific neuronal antibodies such as anti-Homer-3. Timely identification and initiation of immunotherapy may lead to meaningful clinical improvement, underscoring the therapeutic potential of targeted immunomodulatory treatments in autoimmune cerebellar syndromes. ACA associated with Homer-3 antibodies may exhibit a relapsing disease course and is often linked to a poor prognosis, particularly in cases with established cerebellar atrophy. The results of the studies underscore the potential need for early and aggressive immunotherapy, such as rituximab, in managing Homer-3 autoimmunity. Although partial clinical improvement can be achieved, persistent disability may remain. In relapsing or refractory cases, escalation to second line therapies may be required, and long-term immunological monitoring should be considered due to the potential for persistent antibody activity and disease recurrence. Since Homer-3 positive ACA can mimic MSA, it is possible that some cases diagnosed as MSA may be potentially treatable. However, further research is required in this field.

## References

[1] Jarius S Wildemann B. ‘Medusa-head ataxia’: the expanding spectrum of Purkinje cell antibodies in autoimmune cerebellar ataxia. Part 1: Anti-mGluR1, anti-Homer-3, anti-Sj/ITPR1 and anti-CARP VIII. J Neuroinflamm. (2015) 12:166. doi: 10.1186/s12974-015-0356-y, PMID: 26377085 PMC4574226

[2] Hadjivassiliou M Graus F Honnorat J Jarius S Titulaer M Manto M. Diagnostic criteria for primary autoimmune cerebellar ataxia-guidelines from an international task force on immune-mediated cerebellar ataxias. Cerebellum. (2020) 19:605–10. doi: 10.1007/s12311-020-01132-8, PMID: 32328884 PMC7351847

[3] Chien HF Zonta MB Chen J Diaferia G Viana CF Teive HAG. Rehabilitation in patients with cerebellar ataxias. Arq Neuropsiquiatr. (2022) 80:306–15. doi: 10.1590/0004-282X-ANP-2021-0065, PMID: 35239817 PMC9648943

[4] Salman MS. Epidemiology of cerebellar diseases and therapeutic approaches. Cerebellum Lond Engl. (2018) 17:4–11. doi: 10.1007/s12311-017-0885-2, PMID: 28940047

[5] Pedroso JL Vale TC Braga-Neto P Dutra LA França MC Jr Teive HAG. Acute cerebellar ataxia: differential diagnosis and clinical approach. Arq Neuropsiquiatr. (2019) 77:184–93. doi: 10.1590/0004-282X20190020, PMID: 30970132

[6] Liu M Ren H Zhu Y Fan S Bai L Wang J. Autoimmune cerebellar ataxia: etiology and clinical characteristics of a case series from China. Cerebellum. (2023) 22:379–85. doi: 10.1007/s12311-022-01412-5, PMID: 35618871

[7] Kudo A Yaguchi H Tanaka K Kimura A Yabe I. A retrospective study of autoimmune cerebellar ataxia over a 20-year period in a single institution. J Neurol. (2024) 271:553–63. doi: 10.1007/s00415-023-11946-1, PMID: 37610447

[8] Ortiz JF Betté S Tambo W Tao F Cozar JC Isaacson S. Multiple system atrophy - cerebellar type: clinical picture and treatment of an often-overlooked disorder. Cureus. (2020) 12:e10741. doi: 10.7759/cureus.10741, PMID: 33173654 PMC7645310

[9] Ando S Kanazawa M Onodera O. Progressive supranuclear palsy with predominant cerebellar ataxia. J Mov Disord. (2020) 13:20–6. doi: 10.14802/jmd.19061, PMID: 31847511 PMC6987520

[10] Miao A Yu C Sun Y Wang L Ge J Wang X. Acute cerebellitis associated with anti-homer 3 antibodies: A rare case report and literature review. Front Neurol. (2022) 13:837937. doi: 10.3389/fneur.2022.837937, PMID: 35250837 PMC8895947

[11] Klötzsch C Böhmert M Hermann R Teegen B Rentzsch K Till A. Anti-Homer-3 antibodies in cerebrospinal fluid and serum samples from a 58-year-old woman with subacute cerebellar degeneration and diffuse breast adenocarcinoma. Neurol Res Pract. (2022) 4:29. doi: 10.1186/s42466-022-00194-9, PMID: 35871640 PMC9310468

[12] Liu M Ren H Fan S Zhang W Xu Y Zhao W. Neurological autoimmunity associated with homer-3 antibody: A case series from China. Neurol Neuroimmunol Neuroinflamm. (2021) 8:e1077. doi: 10.1212/NXI.0000000000001077, PMID: 34580182 PMC8477375

[13] Fourgeaud L. Addicted to homer? J Neurosci. (2005) 25:9555–6. doi: 10.1523/JNEUROSCI.3492-05.2005, PMID: 16237160 PMC6725735

[14] Shiraishi Y Mizutani A Yuasa S Mikoshiba K Furuichi T. Differential expression of Homer family proteins in the developing mouse brain. J Comp Neurol. (2004) 473:582–99. doi: 10.1002/cne.20116, PMID: 15116392

[15] Ruegsegger C Stucki DM Steiner S Angliker N Radecke J Keller E. Impaired mTORC1-dependent expression of homer-3 influences SCA1 pathophysiology. Neuron. (2016) 89:129–46. doi: 10.1016/j.neuron.2015.11.033, PMID: 26748090

[16] Sakai Y Kassai H Nakayama H Fukaya M Maeda T Nakao K. Hyperactivation of mTORC1 disrupts cellular homeostasis in cerebellar Purkinje cells. Sci Rep. (2019) 9:2799. doi: 10.1038/s41598-019-38730-4, PMID: 30808980 PMC6391425

[17] Guhan N Lu B. Homer-PIKE complex: a novel link between mGluRI and PI 3-kinase. Trends Neurosci. (2004) 27:645–8. doi: 10.1016/j.tins.2004.08.011, PMID: 15474163

[18] Mitoma H Manto M Hadjivassiliou M. Immune-mediated cerebellar ataxias: clinical diagnosis and treatment based on immunological and physiological mechanisms. J Mov Disord. (2021) 14:10–28. doi: 10.14802/jmd.20040, PMID: 33423437 PMC7840241

[19] Höftberger R Sabater L Ortega A Dalmau J Graus F. Patient with homer-3 antibodies and cerebellitis. JAMA Neurol. (2013) 70:506–9. doi: 10.1001/jamaneurol.2013.1955, PMID: 23400636 PMC3723144

[20] Hansen N Radenbach K Rentzsch K Fox J Wiltfang J Bartels C. Cerebrospinal fluid homer-3 autoantibodies in a patient with amnestic mild cognitive impairment. Brain Sci. (2023) 13:125. doi: 10.3390/brainsci13010125, PMID: 36672107 PMC9856294

[21] Zuliani L Sabater L Saiz A Baiges JJ Giometto B Graus F. Homer 3 autoimmunity in subacute idiopathic cerebellar ataxia. Neurology. (2007) 68:239–40. doi: 10.1212/01.wnl.0000251308.79366.f9, PMID: 17224583

[22] Wu Q Gong B Jiang A Qin X. Case report and literature analysis: Autoimmune cerebellar ataxia associated with homer-3 antibodies. Front Neurol. (2022) 13:951659. doi: 10.3389/fneur.2022.951659, PMID: 35959384 PMC9360609

[23] Kuang Z Baizabal-Carvallo JF Mofatteh M Xie S Wang Z Chen Y. Anti-homer-3 antibody encephalitis in a 10-year-old child: case report and review of the literature. Front Neurol. (2022) 13:929778. doi: 10.3389/fneur.2022.929778, PMID: 35769364 PMC9234694

[24] Xu X Ren H Li L Wang J Fechner K Guan H. Anti-Homer-3 antibody associated cerebellar ataxia: A rare case report and literature review. J Neuroimmunol. (2019) 330:155–8. doi: 10.1016/j.jneuroim.2019.01.002, PMID: 30904736

[25] Hu Y Sun Q. Cerebellar swelling followed by atrophy in anti-homer-3 antibody-associated cerebellitis. Neurology. (2022) 99:610–1. doi: 10.1212/WNL.0000000000201105, PMID: 35858816

[26] Klietz M Katzdobler S Levin J Wegner F Höllerhage M Hopfner F. HOMER-3 antibodies were not detected in two German cohorts of patients with multiple system atrophy. Mov Disord. (2022) 37:2165–6. doi: 10.1002/mds.29185, PMID: 35975882

[27] Mulroy E Balint B Bhatiam KP. Homer-3 antibody disease: A potentially treatable MSA-C mimic. Mov. Disord Clin Pract. (2022) 9:178–82. doi: 10.1002/mdc3.13404, PMID: 35146057 PMC8810419

[28] Reshetnikov V Ryabushkina Y Kovner A Lepeshko A Bondar N. Repeated and single maternal separation specifically alter microglial morphology in the prefrontal cortex and neurogenesis in the hippocampus of 15-day-old male mice. Neuroreport. (2020) 31:1256–64. doi: 10.1097/WNR.0000000000001544, PMID: 33165192

[29] Wenning GK Stankovic I Vignatelli L Fanciulli A Calandra-Buonaura G Seppi K. The movement disorder society criteria for the diagnosis of multiple system atrophy. Mov Disord. (2022) 37:1131–48. doi: 10.1002/mds.29005, PMID: 35445419 PMC9321158

[30] Robakis D Louis ED. Head tremor in essential tremor: “Yes-yes”, “no-no”, or “round and round”? Parkinsonism Relat Disord. (2016) 22:98–101. doi: 10.1016/j.parkreldis.2015.11.002, PMID: 26563987 PMC4695227

[31] Paschen S Wolke R Gövert F Lauber A Zeuner KE Helmers AK. Effect of thalamic versus pallidal deep brain stimulation on head tremor in dystonic and essential tremor patients-A retrospective video-blinded study. Mov Disord Clin Pract. (2024) 11:634–44. doi: 10.1002/mdc3.14021, PMID: 38486480 PMC11145156

[32] Mavroudis I Kazis D Petridis F Chatzikonstantinou S Karantali E Njau SN. Morphological and morphometric changes in the Purkinje cells of patients with essential tremor. Exp Ther Med. (2022) 23:167. doi: 10.3892/etm.2021.11090, PMID: 35069848 PMC8753961

[33] Höglinger GU Respondek G Stamelou M Kurz C Josephs KA Lang AE. Clinical diagnosis of progressive supranuclear palsy: The movement disorder society criteria. Mov Disord. (2017) 32:853–64. doi: 10.1002/mds.26987, PMID: 28467028 PMC5516529

[34] Cai Y Hua Z Chen Y Chen X Liu N Liu T. Clinical features of autoimmune cerebellar ataxia related to neuronal antibodies. Front Immunol. (2025) 16:1497695. doi: 10.3389/fimmu.2025, PMID: 40018043 PMC11865250

[35] Jarius S Bräuninger S Chung HY Geis C Haas J Komorowski L. Inositol 1,4,5-trisphosphate receptor type 1 autoantibody (ITPR1-IgG/anti-Sj)-associated autoimmune cerebellar ataxia, encephalitis and peripheral neuropathy: review of the literature. J Neuroinflammation. (2022) 19:196. doi: 10.1186/s12974-022-02545-4, PMID: 35907972 PMC9338677

[36] Zhang W Ren H Ren X Fang F Guan H. Autoimmune cerebellar ataxia associated with anti-Purkinje cells antibodies: the next frontier of neuroimmunology. Ann Transl Med. (2023) 11:285. doi: 10.21037/atm-20-2187, PMID: 37090052 PMC10116421

[37] Assoum M Philippe C Isidor B Perrin L Makrythanasis P Sondheimer N. Autosomal-recessive mutations in AP3B2, adaptor-related protein complex 3 beta 2 subunit, cause an early-onset epileptic encephalopathy with optic atrophy. Am J Hum Genet. (2016) 99:1368–76. doi: 10.1016/j.ajhg.2016.10.009, PMID: 27889060 PMC5142104

[38] Rojas A Wetherington J Shaw R Serrano G Swanger S Dingledine R. Activation of group I metabotropic glutamate receptors potentiates heteromeric kainate receptors. Mol Pharmacol. (2013) 83:106–21. doi: 10.1124/mol.112.081802, PMID: 23066089 PMC3533475

[39] Balint B Vincent A Meinck HM Irani SR Bhatia KP. Movement disorders with neuronal antibodies: syndromic approach, genetic parallels and pathophysiology. Brain. (2018) 141:13–36. doi: 10.1093/brain/awx189, PMID: 29053777 PMC5888977

[40] Garza M Piquet AL. Update in autoimmune movement disorders: newly described antigen targets in autoimmune and paraneoplastic cerebellar ataxia. Front Neurol. (2021) 12:683048. doi: 10.3389/fneur.2021.683048, PMID: 34489848 PMC8416494

[41] Benoit J Muñiz-Castrillo S Vogrig A Farina A Pinto AL Picard G. Early-stage contactin-associated protein-like 2 limbic encephalitis: clues for diagnosis. Neurol Neuroimmunol Neuroinflamm. (2022) 10:e200041. doi: 10.1212/NXI.0000000000200041, PMID: 36288995 PMC9608385

[42] Brouillette AM Öz G Gomez CM. Cerebrospinal fluid biomarkers in spinocerebellar ataxia: A pilot study. Dis Markers. (2015) 2015:413098. doi: 10.1155/2015/413098, PMID: 26265793 PMC4525756

[43] Öz G Hutter D Tkáč I Clark HB Gross MD Jiang H. Neurochemical alterations in spinocerebellar ataxia type 1 and their correlations with clinical status. Mov. Disord. (2010) 25:1253–61. doi: 10.1002/mds.23067, PMID: 20310029 PMC2916651

[44] Hanna Al-Shaikh R Jansen-West K Strongosky A Parrales Z Dunmore JA Song Y. Evaluating glial fibrillary acidic protein and neurofilament light as potential biomarkers for spinocerebellar ataxia 7. Int J Mol Sci. (2025) 26:5070. doi: 10.3390/ijms26115070, PMID: 40507882 PMC12154444

[45] To KW Adamian M Jakobiec FA Berson EL. Olivopontocerebellar atrophy with retinal degeneration. An electroretinographic and histopathologic investigation. Ophthalmology. (1993) 100:15–23. doi: 10.1016/S0161-6420(93)31702-1, PMID: 8433819

[46] Aleman TS Cideciyan AV Volpe NJ Stevanin G Brice A Jacobson SG. Spinocerebellar ataxia type 7 (SCA7) shows a cone-rod dystrophy phenotype. Exp Eye Res. (2002) 74:737–45. doi: 10.1006/exer.2002.1169, PMID: 12126946

[47] Mandel-Brehm C Dubey D Kryzer TJ O’Donovan BD Tran B Vazquez SE. Kelch-like protein 11 antibodies in seminoma-associated paraneoplastic encephalitis. N Engl J Med. (2019) 381:47–54. doi: 10.1056/NEJMoa1816721, PMID: 31269365 PMC6800027

[48] Nanri K Okuma M Sato S Yoneda M Taguchi T Mitoma H. Prevalence of autoantibodies and the efficacy of immunotherapy for autoimmune cerebellar ataxia. Intern Med. (2016) 55:449–54. doi: 10.2169/internalmedicine.55.5156, PMID: 26935362

[49] Giagkou N Stamelou M. Therapeutic management of the overlapping syndromes of atypical Parkinsonism. CNS Drugs. (2018) 32:827–37. doi: 10.1007/s40263-018-0551-3, PMID: 30051337

[50] Madetko N Marzec W Kowalska A Przewodowska D Alster P Koziorowski D. Anti-igLON5 disease - the current state of knowledge and further perspectives. Front Immunol. (2022) 13:852215. doi: 10.3389/fimmu.2022.852215, PMID: 35300333 PMC8921982

[51] Zhang W Xiao D Mao Q Xia H. Role of neuroinflammation in neurodegeneration development. Signal Transduct Target Ther. (2023) 8:267. doi: 10.1038/s41392-023-01486-5, PMID: 37433768 PMC10336149

[52] Garmendia JV De Sanctis CV Das V Annadurai N Hajduch M De Sanctis JB. Inflammation, autoimmunity and neurodegenerative diseases, therapeutics and beyond. Curr Neuropharmacol. (2024) 22:1080–109. doi: 10.2174/1570159X22666231017141636, PMID: 37898823 PMC10964103

[53] Ryding M Gamre M Nissen MS Nilsson AC Okarmus J Poulsen AAE. Neurodegeneration induced by anti-IgLON5 antibodies studied in induced pluripotent stem cell-derived human neurons. Cells. (2021) 10:837. doi: 10.3390/cells10040837, PMID: 33917676 PMC8068068

[54] Sabater L Gaig C Gelpi E Bataller L Lewerenz J Torres-Vega E. A novel non-rapid-eye movement and rapid-eye-movement parasomnia with sleep breathing disorder associated with antibodies to IgLON5: a case series, characterisation of the antigen, and post-mortem study. Lancet Neurol. (2014) 13:575–86. doi: 10.1016/S1474-4422(14)70051-1, PMID: 24703753 PMC4104022

[55] Sabater L Planagumà J Dalmau J Graus F. Cellular investigations with human antibodies associated with the anti-IgLON5 syndrome. J Neuroinflammation. (2016) 13:226. doi: 10.1186/s12974-016-0689-1, PMID: 27586161 PMC5007989

[56] Landa J Gaig C Plagumà J Saiz A Antonell A Sanchez-Valle R. Effects of IgLON5 antibodies on neuronal cytoskeleton: a link between autoimmunity and neurodegeneration. Ann Neurol. (2020) 88:1023–7. doi: 10.1002/ana.25857, PMID: 32740999

[57] Grüter T Möllers FE Tietz A Dargvainiene J Melzer N Heidbreder A. Clinical, serological and genetic predictors of response to immunotherapy in anti-IgLON5 disease. Brain. (2022) 146:600-11. doi: 10.1093/brain/awac090, PMID: 35259208

[58] Zhang YH Ni Y Gao YN Shen DD He L Yin D. Anti-IgLON5 disease: a novel topic beyond neuroimmunology. Neural Regener Res. (2023) 18:1017–22. doi: 10.4103/1673-5374.355742, PMID: 36254983 PMC9827781

[59] Brzecka A Leszek J Ashraf GM Ejma M Ávila-Rodriguez MF Yarla NS. Sleep disorders associated with Alzheimer’s disease: A perspective. Front Neurosci. (2018) 12:330. doi: 10.3389/fnins.2018.00330, PMID: 29904334 PMC5990625

[60] Tahami Monfared AA Phan NTN Pearson I Mauskopf J Cho M Zhang Q. A systematic review of clinical practice guidelines for Alzheimer’s disease and strategies for future advancements. Neurol Ther. (2023) 12:1257–84. doi: 10.1007/s40120-023-00504-6, PMID: 37261607 PMC10310649

[61] Armstrong MJ Litvan I Lang AE Bak TH Bhatia KP Borroni B. Criteria for the diagnosis of corticobasal degeneration. Neurology. (2013) 80:496–503. doi: 10.1212/WNL.0b013e31827f0fd1, PMID: 23359374 PMC3590050

[62] Arendt T Stieler JT Holzer M. Tau and tauopathies. Brain Res Bull. (2016) 126:238–92. doi: 10.1016/j.brainresbull.2016.08.018, PMID: 27615390

[63] Alster P Madetko-Alster N Migda A Migda B Kutyłowski M Królicki L. Sleep disturbances in progressive supranuclear palsy syndrome (PSPS) and corticobasal syndrome (CBS). Neurol Neurochir Pol. (2023) 57:229–34. doi: 10.5603/PJNNS.a2023.0019, PMID: 36928793

